# A Novel Minimally Invasive Porcine Model of Functional Tricuspid Regurgitation

**DOI:** 10.3390/jcdd13040166

**Published:** 2026-04-14

**Authors:** Claudia González-Cucharero, Ignacio Hernández, Javier Díez-Mata, Rafael Ramírez-Carracedo, Marta Saura, Claudia Baéz-Díaz, Fátima Vázquez-López, Francisco M. Sánchez-Margallo, Jose L. Zamorano, Verónica Crisóstomo, Carlos Zaragoza

**Affiliations:** 1 Unidad Mixta de Investigación Cardiovascular Universidad Francisco de Vitoria, Hospital Universitario Ramón y Cajal (IRYCIS), 28034 Madrid, Spain; claudia.gonzalezcuch@edu.uah.es (C.G.-C.); ignacio.hernandez@ufv.es (I.H.); javier.diez@ufv.es (J.D.-M.); 2 Centro de Investigación Biomédica en Red de Enfermedades Cardiovasculares (CIBERCV), Instituto de Salud Carlos III (ISCIII), 28029 Madrid, Spain; marta.saura@uah.es (M.S.); zamorano@secardiologia.es (J.L.Z.); 3 Departamento de Cirugía Ciencias Medicas y Sociales, Universidad de Alcalá, Alcalá de Henares, 28872 Madrid, Spain; rafael.ramirez@uah.es; 4 Unidad de Fisiología, Departamento de Biología de Sistemas, Universidad de Alcalá (IRYCIS), Alcalá de Henares, 28871 Madrid, Spain; 5 Fundación Centro de Cirugía Mínimamente Invasiva Jesús Usón, 10004 Cáceres, Spain; cbaez@ccmijesususon.com (C.B.-D.); fvazquez@ccmijesususon.com (F.V.-L.); msanchez@ccmijesususon.com (F.M.S.-M.); 6 Red Española de Terapias Avanzadas RICORS-TERAV, 28029 Madrid, Spain; 7 Departamento de Cardiología, Hospital Universitario Ramón y Cajal (IRYCIS), 28034 Madrid, Spain

**Keywords:** tricuspid regurgitation, porcine model, atrial fibrilation, heart failure

## Abstract

Tricuspid regurgitation (TR) is a prevalent cardiovascular disorder with significant clinical impact. TR is frequently silent and underdiagnosed and is estimated to impact over 70 million people globally. Characterized by retrograde blood flow from the right ventricle into the right atrium due to incomplete valve closure, TR leads to right heart dilation, systemic congestion, and eventually right-sided heart failure. Importantly, TR may contribute to the onset of atrial fibrillation (AF), the most common sustained arrhythmia, affecting approximately 59 million individuals worldwide. Despite its growing clinical importance, the pathophysiology of TR remains incompletely understood, and current animal models of TR, based on direct valve manipulation, limit translational applicability. We present a novel, minimally invasive porcine model of TR established via femoral/jugular vein catheterization with deployment of an inferior vena cava (IVC) filter. The filter partially impedes tricuspid valve closure, inducing TR without valvular injury. Validation was achieved through multimodal imaging, including fluoroscopy, echocardiography, and electrocardiography, confirming hallmark features of TR, including right atrial and ventricular enlargement and arrhythmic activity. This model provides a reproducible, minimally invasive platform for studying selected features of TR progression. Its minimally invasive nature and preservation of native valvular structure make it a useful preclinical platform for mechanistic and translational research.

## 1. Introduction

Tricuspid regurgitation (TR) is an increasingly prevalent valvular heart disease that has long been underestimated in clinical practice [1,2]. Once considered a benign condition or a consequence of left-sided heart disease, growing evidence has demonstrated that moderate-to-severe TR is independently associated with increased morbidity and recurrent hospitalizations [3,4]. The pathophysiology of TR is complex and multifactorial, involving dynamic interactions between the tricuspid valve leaflets, annulus, right ventricle and right atrium [5]. Progressive right ventricular dilation, altered ventricular geometry, papillary muscle displacement, leaflet tethering and annular remodeling contribute to impaired leaflet coaptation and progressive blood regurgitation [6,7,8]. Despite growing identification of these mechanisms, the contribution of each and their temporal sequence during disease development remains incompletely understood [9].

In recent years, the clinical relevance of TR has gained renewed attention due to population aging and improved survival. In parallel, major advances in transcatheter tricuspid valve therapies, such as edge-to-edge repair, annuloplasty and valve replacement, have expanded therapeutic options and are gradually improving symptoms and quality of life in high-risk patients [10,11,12,13]. However, despite these technological advances, treatment strategies remain limited in early disease stages or in patients who are not candidates for intervention [14,15,16].

Advancing the understanding of TR and developing effective therapeutic strategies require robust experimental platforms that allow controlled induction of regurgitation, evaluation of disease progression and detailed hemodynamic and structural characterization [17]. Several animal models of TR have been described using different species, including ovine, canine and porcine models [18,19,20,21]. However, all these approaches have typically relied on open-chest surgery, cardiopulmonary bypass or direct leaflet damage to induce regurgitation. While such models have contributed valuable insights, they present important limitations that reduce their translational applicability.

This study aimed to develop a minimally invasive porcine model of tricuspid regurgitation preserving native valvular integrity. Inferior vena cava filter deployment reproducibly induced right-sided chamber dilation, regurgitant flow, and arrhythmic activity, providing a translational platform for mechanistic and therapeutic studies.

## 2. Materials and Methods

### 2.1. Ethical Approval

All experimental procedures were approved by the Ethics Committee of the Jesús Usón Minimally Invasive Surgery Centre (Cáceres, Spain; CCMIJU: 025/24, approved 3 December 2024) and by the Competent Authority (Extremadura Regional Government; EXP-20250318, approved 21 March 2025). All experiments were conducted in accordance with the Guide for the Care and Use of Laboratory Animals, European Animal Research Association. EU regulations on animal research, complying with the 3Rs (replacement, reduction and refinement).

### 2.2. Animal Model and Preoperative Preparation

A total of six pigs were included in the study and followed for 8 weeks. Pigs weighing 50–60 kg were used in this study. Animals were fasted from food and water for at least 5 h prior to the procedure. On the day of surgery, intramuscular sedation was administered using midazolam (0.5 mg/kg, Pfizer, Madrid, Spain), ketamine (10 mg/kg, Pfizer, Madrid, Spain) and xylazine (5 mg/kg, Pfizer, Madrid, Spain). After a 10–15 min induction period, an 18-G intravenous cannula was placed in the marginal ear vein to establish vascular access. Orotracheal intubation was performed using a 7.5 mm internal diameter endotracheal tube appropriate for the animal weight range.

### 2.3. Anesthesia and Intraoperative Monitoring

Upon arrival in the catheterization laboratory, mechanical ventilation with positive pressure was initiated using a fraction of inspired oxygen (FiO_2_) of 0.50 and a tidal volume of 12 mL/kg. Balanced anesthesia was maintained with a continuous intravenous infusion of fentanyl (3–5 µg/kg/h, Kern Pharma (Madrid, Spain) combined with inhalational anesthesia using sevoflurane (Abbvie (North Chicago, IL, USA) at a concentration of 2.5%.

Anesthetic depth was assessed by evaluation of corneal reflexes and respiratory pattern, with the presence of spontaneous breathing considered indicative of insufficient anesthesia. Veterinary lubricating ophthalmic ointment (Visufarma, Madrid, Spain) was applied to both eyes to prevent corneal dryness. Continuous heart rate monitoring was performed using electrocardiography.

### 2.4. Echocardiographic Assessment

Transthoracic echocardiography (TTE) was performed using a commercially available ultrasound system (BK5000) (GE Healthcare, Madrid, Spain) equipped with a GE 5P1 small-footprint cardiac transducer (frequency range 5–1 MHz). Standard two-dimensional, M-mode, color Doppler, pulsed-wave Doppler, and continuous-wave Doppler images were attempted and acquired when feasible, from parasternal and subcostal acoustic windows at baseline and during weekly follow-up.

Two-dimensional and M-mode imaging were used for general cardiac anatomical and morphological assessment. For tricuspid regurgitation evaluation, both parasternal and subcostal windows were explored in each animal, and the view with the best achievable alignment to the regurgitant jet was selected for Doppler interrogation. Because of the anatomical and acoustic constraints of transthoracic imaging in pigs, Doppler data were interpreted mainly in a qualitative/semi-quantitative manner and integrated with color Doppler jet visualization, serial chamber measurements, and fluoroscopic confirmation of device position.

Continuous-wave (CW) Doppler and M-mode indices were acquired when feasible; however, given their dependence on reproducible optimal acoustic windows and near-parallel Doppler alignment, they were not prespecified as primary longitudinal quantitative endpoints. Vena contracta (VC) width was initially measured as an exploratory parameter, but it was not included in the final quantitative analysis because the device-mediated “valve-held-open” configuration and plane dependency limited the robustness of 2D VC for standardized serial quantification.

For longitudinal quantitative assessment of right-heart remodeling, right atrial and right ventricular chamber areas were measured from two-dimensional images at end-diastole and end-systole using the best achievable imaging plane at each time point. These area-based measurements were used as the main echocardiographic parameters for serial follow-up of right-sided chamber dilation.

### 2.5. Percutaneous Central Venous Access

Under ultrasound guidance, central venous access was obtained using either a jugular or femoral approach, depending on procedural requirements. For the jugular approach, the jugular–carotid vascular bundle was first identified, and the jugular vein was cannulated using a 7 French introducer catheter with its dilator, following insertion of a 0.035-inch guidewire. Alternatively, when a femoral approach was selected, the femoral vein was identified under ultrasound guidance and cannulated using a vascular trocar. A 0.035-inch guidewire was then advanced toward the right ventricle.

Once correct intravascular positioning was confirmed for either access route by injecting contrast medium and ensuring accurate localization within the target vascular territory, the dilator and the 0.035-inch guidewire were removed, leaving the introducer in place to allow subsequent catheter-based measurements and inferior vena cava (IVC) filter delivery.

### 2.6. Hemodynamic Procedure and IVC Filter Deployment

Under fluoroscopic guidance, a 0.035-inch hydrophilic guidewire advanced through the jugular/femoral 7 French introducer and across the tricuspid valve. Transient ventricular ectopic beats were observed during valve crossing due to mechanical contact between the guidewire and the endocardium. The guidewire was further advanced into the main pulmonary artery to serve as a fluoroscopic and anatomical reference.

An inferior vena cava filter delivery system (Option Elite, Argon Medical, Madrid, Spain), was then advanced over the 0.035-guidewire between the right atrium and ventricle. Using the support provided by the guidewire, the entire device was carefully advanced through the right atrium into the right ventricle, ensuring that the distal portion of the system was positioned just beyond the tricuspid valve. Once optimal positioning was achieved, the guidewire was carefully withdrawn to prevent system displacement and facilitate device deployment.

Using a curved pusher, the filter was centered and aligned with the tricuspid valve. The delivery sheath was gradually withdrawn, allowing slow and symmetric expansion of the filter. After complete deployment (Supplementary Figure S1), the delivery system was fully removed, and correct anchoring of the IVC filter at the tricuspid valve level was confirmed by fluoroscope, as shown in a real-time video recording (Supplementary Figure S2).

### 2.7. Anesthesia Reversal and Postoperative Recovery

All anesthetic agents, including inhaled sevoflurane and intravenous fentanyl, were discontinued. Mechanical ventilation was gradually reduced to assess spontaneous respiration. Once adequate spontaneous breathing and hemodynamic stability were confirmed, the animal was extubated and provided with supplemental oxygen via face mask. Animals were placed in sternal recumbency in a warm, quiet recovery area until full recovery of consciousness and mobility. Postoperative analgesia was administered in accordance with veterinary ethical standards.

### 2.8. Statistical Analysis

Data analysis was performed using GraphPad Prism software 8.0 (GraphPad Software, San Diego, CA, USA). Continuous variables are presented as mean ± standard deviation or median, as appropriate. Comparisons between time points or experimental conditions were performed using paired or unpaired Student’s *t*-tests or non-parametric equivalents when normality assumptions were not met. A two-tailed *p* value < 0.05 was considered statistically significant.

## 3. Results

### 3.1. IVC Implementation and Validation Through Cardiac Fluoroscopy Imaging

The minimally invasive porcine model of TR developed via deployment of an inferior vena cava (IVC) filter was successfully implemented. Proper placement of the filter across the tricuspid valve annulus was confirmed by real-time fluoroscopy, ensuring consistent mechanical interference with leaflet coaptation and sustained regurgitation throughout follow-up.

**Figure 1:**
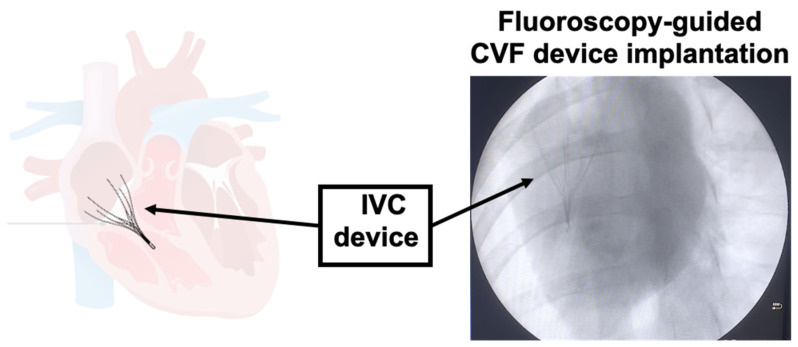
IVC device implantation.

Fluoroscopy was an essential tool in confirming the accurate deployment during implantation at the tricuspid annular level and in reliably confirming IVC filter positioning. Immediately after transjugular insertion, the IVC filter was visualized across the tricuspid valve during cardiac cycles, demonstrating effective mechanical interaction with the valve in all successful cases. No evidence of device migration, collapse, or embolization was detected during the 8-week observational period (Figure 1).

#### Echocardiographic Validation

Echocardiography is the primary tool for the detection and grading of tricuspid regurgitation in the porcine model. Several evaluations are performed at baseline and weekly post-procedure, confirming both the correct implantation of the IVC filter and demonstrating features of TR in patients. The device remained centrally positioned across the tricuspid valve without displacement throughout the eight-week follow-up period. No evidence of device relocation, structural collapse, or filter embolization was detected.

**Figure 2:**
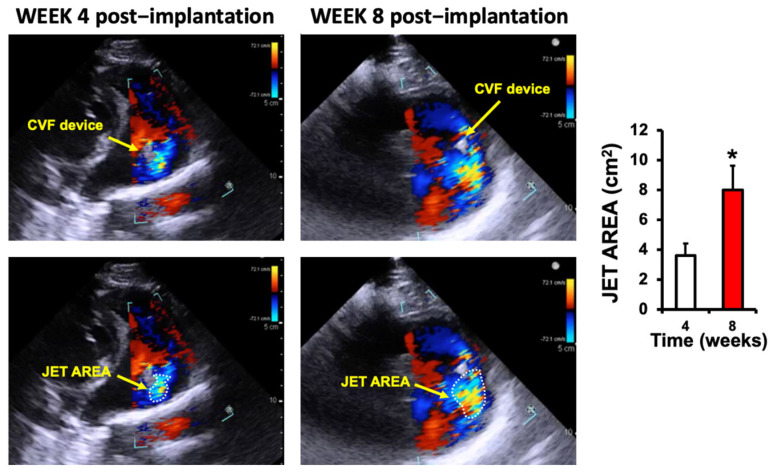
Echocardiographic assessment of regurgitant jet area in animals implanted with a cava vein filter (CVF). Representative color Doppler echocardiographic images showing the position of the CVF device (upper panels) and the corresponding regurgitant jet area outlined in dotted lines (lower panels), at 4 and 8 weeks post−implantation, as indicated. The bar graph (**right**) summarizes the mean jet area (mean ± SD, *n* = 6) measured at 4 and 8 weeks post−CVF implantation. A significant increase in jet area was observed at 8 weeks compared to 4 weeks. (* *p* < 0.02 at week 8 vs. week 4).

Color Doppler demonstrated regurgitant jets within the first 7 days, with regurgitant jet area increased from 3.6 ± 0.8 cm^2^ in the fourth week to 8.0 ± 1.6 cm^2^ at 8 weeks (*p* < 0.05) (Figure 2).

**Figure 3:**
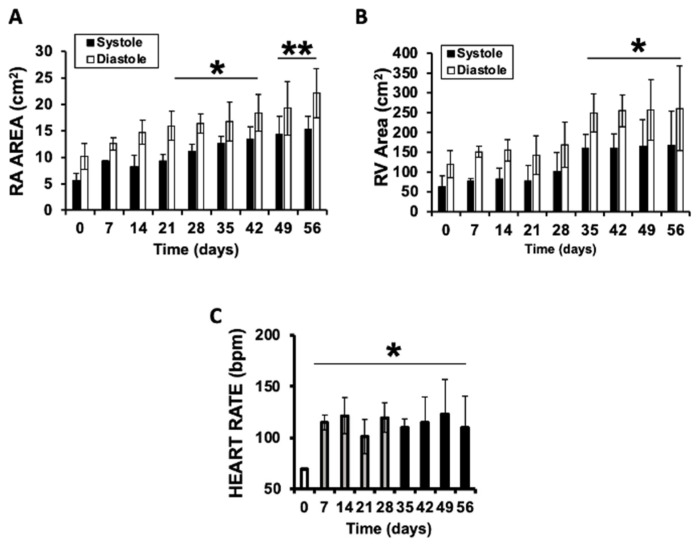
Longitudinal echocardiographic assessment of right heart chamber areas and heart rate in animals implanted with an inferior vena cava (IVC) filter. (**A**) Right atrial end-diastolic (white bars) and end-systolic (black bars) areas. (**B**) Right ventricular end-diastolic (white bars) and end-systolic (black bars) areas. (**C**) Heart rate (beats per minute) measured at each time point. (*n* = 6, weekly assayed. Values were expressed as mean ± SD, (**A**) * *p* < 0.03 diastolic 21–42 days vs. diastolic 0 days, ** *p* < 0.05 diastolic 49, and 56 days vs. diastolic 0 days. (**B**) * *p* <0.05 diastolic 35–56 days, vs. diastolic 0 days. (**C**) * *p* < 0.01 7–56 days vs. 0 days).

Chamber remodeling was evident by progressive enlargement of the right atrium, which increased significantly both in systole, 5.6 ± 1.9 cm^2^ vs. 22.1 ± 6.5 cm^2^, and diastole, 10.1 ± 3.2 cm^2^ vs. 22.1 ± 5.8 cm^2^ (Figure 3A). Similarly, the right ventricular chamber showed marked dilation, with systolic area increasing from 62.6.0 ± 29.5 cm^2^ to 167.0 ± 63.0 cm^2^, and end-diastolic area from 119.1 ± 28.9 cm^2^ to 261 ± 58.7 cm^2^ (Figure 3B). Additionally, heart rate increased in the first week after implantation, from 65 ± 8 bpm at baseline to 127 ± 19 bpm by week eight, consistent with progressive right heart burden (Figure 3C). Taken together, these changes support the successful induction of chronic tricuspid regurgitation associated with progressive right-heart chamber dilation within the initial eight weeks of the model.

**Figure 4:**
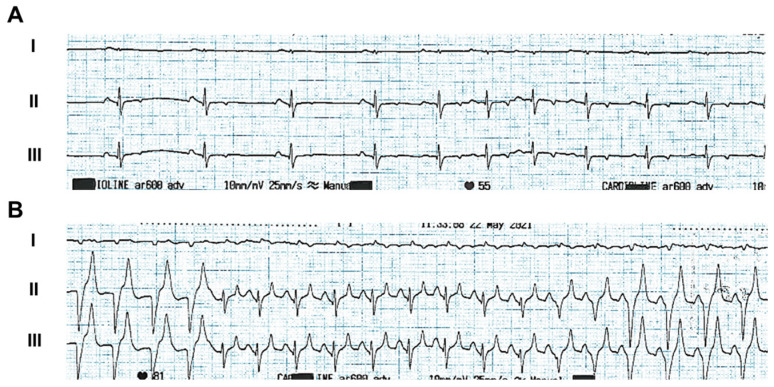
Electrocardiographic detection of arrhythmogenic events in pigs after IVC implantation. Representative electrocardiographic traces from two independent events recorded 2 months after IVC implantation: (**A**) multifocal atrial tachycardia with irregular R–R intervals and (**B**) non-sustained ventricular tachycardia.

Electrocardiographic monitoring revealed arrhythmogenic activity in animals implanted with the inferior vena cava filter. At eight weeks post-implantation, recordings showed multifocal atrial tachycardia with irregular R–R intervals and increased P-wave amplitude, consistent with atrial overload secondary to tricuspid dysfunction (Figure 4A). Episodes of non-sustained ventricular tachycardia were also observed (Figure 4B). These abnormalities were absent at baseline and developed in parallel with chamber remodeling.

## 4. Discussion

The protocol presented describes the development of a minimally invasive porcine model of TR using the deployment of an inferior vena cava filter across the tricuspid valve. Several technical steps are critical to ensure reproducibility. First, accurate fluoroscopic guidance during IVC filter routing and the exact place of filter deployment is essential to guarantee stable attachment without migration. In addition, echocardiographic validation is required both at baseline and during follow-up to confirm regurgitant blood flow and to monitor progressive chamber dilation. Electrocardiogram follow-up is also important, as it provides important information about P wave morphology.

Potential modifications of the method include the use of different filter sizes or alternative transvenous approaches. In addition, modulate the opening of IVC filter may help adjusting regurgitation severity. These variations will be translated into differences in hemodynamic impact and remodeling outcomes, allowing the model to better replicate diverse clinical scenarios. Regarding troubleshooting, it commonly involves preventing filter malposition, which can be minimized by slow and controlled withdrawal during deployment with the fluoroscopic imaging.

As with other experimental models of tricuspid regurgitation, the present approach does not reproduce the full etiological complexity of the human disease, but rather selected features that are relevant to the intended experimental question. Traditional approaches rely on invasive surgical techniques [22], including chordal cutting or radiofrequency ablation, which offer anatomical control but are often invasive, slow to induce pathology, and poorly reflective of functional human TR [21,23]. Mechanical models such as pulmonary artery banding or annular damage can reproduce right-sided overload, whereas pharmacological approaches, such as monocrotaline-induced pulmonary hypertension, offer a simpler alternative but primarily simulate functional TR without accurately reflecting its multifactorial nature. Additionally, percutaneous models used for device evaluation do not necessarily mimic disease physiology [24]. Hybrid models [25] combining multiple triggers (volume, electrical, and structural stress) have improved relevance but remain technically complex, costly, and difficult to replicate consistently. In this context, our model should be understood as a minimally invasive device-induced model of tricuspid regurgitation that preserves native valvular integrity and reproducibly generates a chronic regurgitant state followed by progressive right atrial and right ventricular remodeling

Human functional tricuspid regurgitation is a heterogeneous entity that most commonly arises from atrial and/or right ventricular remodeling, leading to progressive annular dilation, reduced leaflet coaptation and, in many cases, leaflet tethering, often in the context of atrial fibrillation and/or pulmonary hypertension [7,9,16]. In contrast, in the present model TR is initiated by device-mediated prevention of leaflet coaptation (“valve-held-open” configuration) and therefore does not reproduce the full etiological cascade or geometric substrate that drives human FTR. Accordingly, this model should not be interpreted as a mechanistic replica of specific FTR subtypes (atrial vs. ventricular FTR) nor as a platform to study the upstream determinants of annular dilation and tethering. Because follow-up was limited to eight weeks and designed primarily for proof-of-concept phenotypic validation, we cannot determine whether longer-term exposure to sustained TR in this model may secondarily drive annular dilation and/or tethering patterns that more closely resemble clinical FTR phenotypes.

Importantly, however, once established, the model generates a sustained regurgitant lesion with chronic right-sided volume overload and progressive right-heart remodeling during follow-up, which represent central downstream consequences of clinically relevant TR. Thus, the model is particularly suited for proof-of-concept phenotypic validation and standardized longitudinal assessments in a controlled TR environment (e.g., feasibility/durability testing of transcatheter concepts), while complementary models may be preferable when the primary aim is to investigate the etiopathogenesis of human FTR or detailed annular/leaflet biomechanics.

Given these limitations, we developed a novel, minimally invasive porcine model of functional TR based on the transvenous deployment of a retrievable inferior vena cava filter. By mechanically interfering with tricuspid valve closure, this approach induces reproducible regurgitation without direct leaflet injury, leading to progressive right-sided dilation, atrial and ventricular remodeling, and the onset of arrhythmic behavior. Validation through fluoroscopy, echocardiography and electrocardiography confirmed the development of key TR features, including chamber dilation and regurgitant jets.

This model offers several advantages over traditional approaches. It avoids direct surgical intervention while minimizing animal distress. Another major advantage of this protocol is its use of a large-animal model, specifically the pig, whose cardiac anatomy and physiology, including heart size, chamber geometry and conduction system, closely resemble humans’ heart [26,27,28]. This similarity greatly enhances translational value, as findings regarding remodeling and fibrosis can be more directly extrapolated to patients. Its reproducibility with human disease makes it especially valuable for studying pathophysiological mechanisms, validating biomarkers, and testing therapeutic devices or pharmacological strategies [29]. In addition, it provides the opportunity for studying pathology mechanisms, including the interplay between right atrial dilation, fibrosis and other possible associated disorders.

Swine are a highly valuable large-animal model, yet important anatomical/topographic differences should be considered when translating procedural steps and imaging guidance to humans, as these can influence echocardiographic acquisition and may also influence catheter orientation during fluoroscopy-guided procedures. However, after taking these minor adjustments into account, they do not detract from the model’s utility; rather, they provide essential context for reproducibility and planning translational studies [30].

This study should be considered a first proof-of-concept step aimed at determining whether a minimally invasive transcatheter approach could reproducibly generate the phenotype of functional tricuspid regurgitation in a porcine model. At this stage, the study was primarily focused on demonstrating procedural feasibility and confirming phenotype development by fluoroscopy, serial echocardiography, and electrocardiographic follow-up. The next step will be to incorporate invasive hemodynamic assessment before and immediately after device implantation, as well as during follow-up, in order to achieve a more complete physiological characterization of the model.

Despite its advantages, this model has certain limitations. The IVC filter induces regurgitation through mechanical interference rather than replicating intrinsic leaflet or annular pathology. As a result, the model does not reproduce all etiological subtypes of TR. In addition, invasive hemodynamic measurements were not systematically obtained; therefore, the present study does not provide a direct pressure-based characterization of the acute physiological effects of filter deployment. Echocardiographic feasibility also represented a limitation of this proof-of-concept study. CW Doppler and M-mode indices (including TAPSE) were acquired when feasible but were not consistently reproducible during serial transthoracic examinations in anesthetized, intubated swine, mainly due to non-reproducible acoustic windows, particularly apical views, and the strong dependence of CW Doppler on near-parallel alignment with the regurgitant jet. Moreover, although vena contracta (VC) was initially measured, the device-mediated “valve-held-open” configuration and plane dependency of 2D color Doppler limited the robustness of 2D VC for standardized longitudinal quantification; therefore, VC was not included in the final analysis. Furthermore, long-term follow-up beyond eight weeks has not yet been assessed. This is particularly relevant because tricuspid regurgitation can lead to arrhythmias over longer time frames, and the absence of extended observation may prevent the identification of associations with these secondary pathologies. A further limitation is the relatively small sample size inherent to large-animal longitudinal studies, which may limit the precision and generalizability of quantitative estimates. Therefore, results should be interpreted as proof-of-concept and reproducibility of the model rather than definitive effect sizing. Finally, another limitation is the lack of a separate untreated or sham-operated control group. In this proof-of-concept study, each animal served as its own baseline control through longitudinal pre- and post-implantation assessment. This design helped us observe progressive structural and functional changes post-implantation, but future studies should include sham or untreated animals to better rule out confounding factors, including anesthesia, catheterization, and repeated imaging. Additionally, as in all large-animal studies, costs and ethical considerations must be considered.

The present follow-up was limited to eight weeks, and the rapid right-heart remodeling observed in this experimentally induced model should not be interpreted as directly reproducing the temporal progression of human secondary tricuspid regurgitation. Rather, it may reflect a compressed experimental time course suitable for proof-of-concept phenotypic validation. Future studies should incorporate longer follow-up and circulating biomarkers, such as natriuretic peptides, and implement standardized acquisition strategies (e.g., structured subxiphoid interrogation and, when feasible, complementary modalities) to improve Doppler feasibility and enable a more comprehensive multiparametric grading of TR to provide a more complete biological characterization of the model.

## 5. Conclusions

This minimally invasive porcine model provides a reproducible platform for inducing tricuspid regurgitation and studying its associated right-heart remodeling in a translationally relevant large-animal setting. The model may be useful for investigating disease mechanisms and for evaluating emerging transcatheter, electrophysiological, and pharmacological interventions. Further studies incorporating longer follow-up and broader physiological characterization will be important to define its full translational scope.

In summary, this minimally invasive approach provides a reproducible and translationally relevant tool to bridge the gap between basic cardiovascular research and clinical application.

## Data Availability

Data will be available upon a permission request to the Corresponding Authors of the manuscript.

## Supplementary Materials

The following supporting information can be downloaded at https://www.mdpi.com/article/10.3390/jcdd13040166/s1: Supplementary Figure S1. Real-time video recording of IVC filter implantation and deployment in the heart; Supplementary Figure S2. Real-time video recording of the IVC filter anchored in the heart.
