# Supplementary material for: A Novel Minimally Invasive Porcine Model of Functional Tricuspid Regurgitation
Source: J Cardiovasc Dev Dis. 2026 Apr 14;13(4):166. doi: 10.3390/jcdd13040166 (PMC13116767; doi:10.3390/jcdd13040166)

Supplementary Figure S1. Real-time video recording of IVC filter implantation and deployment in the heart.

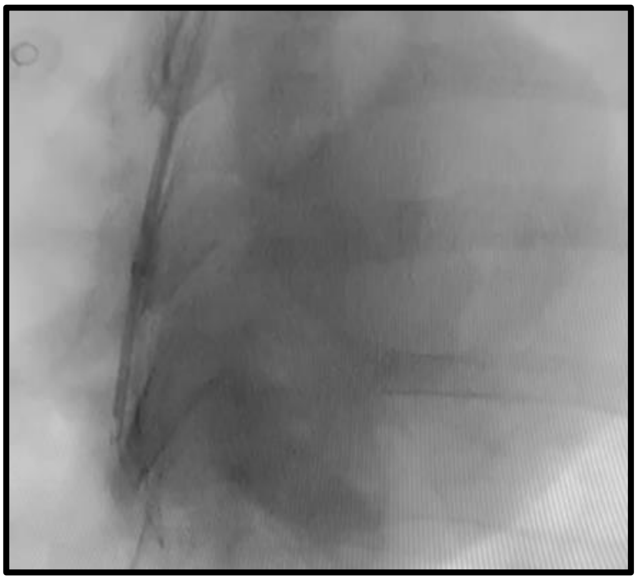

Supplementary Figure S2. Real-time video recording of the IVC filter anchored in the heart

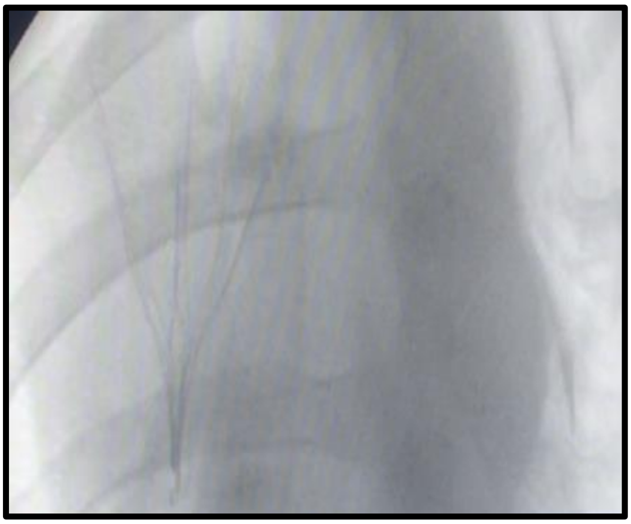

Supplement: Supplementary file 1 [file jcdd-13-00166-s001.zip › jcdd-4142457-supplementary.pdf]
